# Chromosomal diversification and karyotype evolution of diploids in the cytologically diverse genus *Prospero* (Hyacinthaceae)

**DOI:** 10.1186/1471-2148-13-136

**Published:** 2013-07-03

**Authors:** Tae-Soo Jang, Khatere Emadzade, John Parker, Eva M Temsch, Andrew R Leitch, Franz Speta, Hanna Weiss-Schneeweiss

**Affiliations:** 1 Department of Systematic and Evolutionary Botany, University of Vienna, Rennweg 14, A-1030, Vienna, Austria; 2 Cambridge University Botanic Garden, Cambridge CB2 1JF, UK; 3 Queen Mary College, University of London, London, UK; 4 Dornacher Strasse 1, Linz 4040, Austria

**Keywords:** Chromosomal evolution, FISH, Genome size, Hyacinthaceae, ITS, Phylogeny, Prospero, rDNA

## Abstract

**Background:**

*Prospero* (Hyacinthaceae) provides a unique system to assess the impact of genome rearrangements on plant diversification and evolution. The genus exhibits remarkable chromosomal variation but very little morphological differentiation. Basic numbers of *x* = 4, 5, 6 and 7, extensive polyploidy, and numerous polymorphic chromosome variants were described, but only three species are commonly recognized: *P. obtusifolium*, *P. hanburyi*, and *P. autumnale* s.l., the latter comprising four diploid cytotypes. The relationship between evolutionary patterns and chromosomal variation in diploids, the basic modules of the extensive cytological diversity, is presented.

**Results:**

Evolutionary inferences were derived from fluorescence *in situ* hybridization (FISH) with 5S and 35S rDNA, genome size estimations, and phylogenetic analyses of internal transcribed spacer (ITS) of 35S rDNA of 49 diploids in the three species and all cytotypes of *P. autumnale* s.l. All species and cytotypes possess a single 35S rDNA locus, interstitial except in *P. hanburyi* where it is sub-terminal, and one or two 5S rDNA loci (occasionally a third in *P. obtusifolium*) at fixed locations. The localization of the two rDNA types is unique for each species and cytotype. Phylogenetic data in the *P. autumnale* complex enable tracing of the evolution of rDNA loci, genome size, and direction of chromosomal fusions: mixed descending dysploidy of *x* = 7 to *x* = 6 and independently to *x* = 5, rather than successive descending dysploidy, is proposed.

**Conclusions:**

All diploid cytotypes are recovered as well-defined evolutionary lineages. The cytogenetic and phylogenetic approaches have provided excellent phylogenetic markers to infer the direction of chromosomal change in *Prospero*. Evolution in *Prospero*, especially in the *P. autumnale* complex, has been driven by differentiation of an ancestral karyotype largely unaccompanied by morphological change. These new results provide a framework for detailed analyses of various types of chromosomal rearrangements and karyotypic variation in polyploids.

## Background

Chromosomal change plays an important role in plant evolution, diversification, and speciation [1,2]. When carried out against a phylogenetic background [1,3-5] comparative analyses of karyotypes allow inferences regarding evolutionary history.

Detailed physical chromosomal maps, which enable evolutionary patterns and processes to be determined, can be constructed using FISH (fluorescence *in situ* hybridization) from both single copy and repetitive DNAs, such as rDNA, species- or genus-specific repetitive DNAs, individual chromosome DNAs [1,6-10]. Patterns of chromosomal evolution using FISH have been established in several economically important plant genera (e.g., *Nicotiana*[3,11], *Beta*[12]) as well as in model organisms and their wild relatives (e.g., Brassicaceae [1,13]). Comparative evolutionary cytogenetics of wild plant groups, however, has been much less explored (e.g., *Hepatica*[14], *Anemone*[15], *Melampodium*[16]).

The markers of choice for cytogenetic evolutionary studies include tandemly repeated genes encoding 5S and 35S rRNA within the nucleus. The 35S rDNA loci (18S–5.8S–25S rDNA) are located in the nucleolar-organizer regions (NORs), while tandem arrays of 5S rDNA map independently of them (but see [17]). Copy numbers of 5S rDNA are usually lower than 35S rDNA [18,19]. Since the coding regions of these two markers are conserved across large evolutionary units [4,20] their localization provides useful landmarks for chromosome identification [20-22]. Partial DNA sequences of these two rDNA types (e.g., ITS of 35S rDNA or NTS of 5S rDNA) are also commonly used for inferring phylogenies [16,23]. This allows the interpretation of cytological information in a strict phylogenetic context, giving detailed insights into the patterns of evolution of genomes.

**Figure 1:**
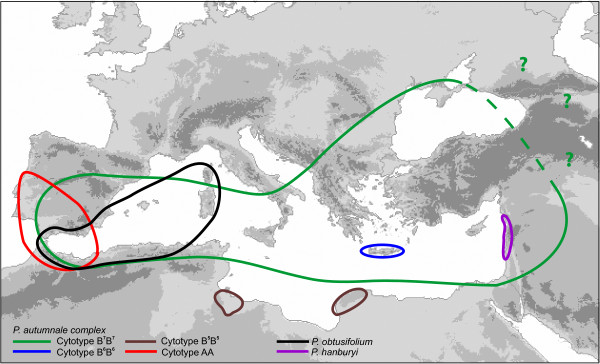
**Map of distribution of diploid species and cytotypes of the genus *Prospero*.** Dashed line in the eastern range of distribution of cytotype B^7^B^7^ and question marks indicate incomplete information on the distribution of this cytotype.

A particularly suitable system for analyzing the role of chromosomal change in plant diversification and speciation is provided by the genus *Prospero* Salisb. (Hyacinthaceae). This genus is distributed around the whole Mediterranean basin, north to Britain and Russia (Figure 1). Across this area *Prospero* exhibits exceptionally high levels of chromosomal variation, with basic chromosome numbers of *x* = 4, 5, 6, and 7, alongside levels of ploidy up to about 20-fold [24-26]. Three species are commonly recognized in the genus: *P*. *obtusifolium* (Poir.) Speta (*x* = 4) and *P. hanburyi* (Baker) Speta (*x* = 7), both chromosomally stable, and a dynamic species complex referred to as *P*. *autumnale* (L.) Speta. Within *P. autumnale,* up to 15 smaller, local, segregates have been described [27-33], but these are only subtly differentiated morphologically (quantitative differences and distinct chromosome numbers/ploidy levels, [32]). Thus, in this paper, we recognize only the three species as comprising *Prospero* for the clarity of the data interpretation. The relationship of genomic, chromosomal, and phylogenetic analyses to species delimitation and their correlation with distinct morphological characters will only emerge from broader evolutionary studies of the genus.

*P. obtusifolium* (*x* = 4) and *P. hanburyi* (*x* = 7) are morphologically distinct entities found within the range of the *P. autumnale* complex, the former two being geographically restricted to the western Mediterranean and to the Levant respectively. They are known only as diploids. By contrast, the *P*. *autumnale* complex exhibits a spectacular array of genomic and chromosomal variation, unparalleled in any other flowering plant, with multiple basic chromosome numbers, a huge range of levels and complexity of polyploidy, and a spectacular array of chromosomal polymorphisms (including supernumerary segments, B-chromosomes, and inversions). Four distinct diploid cytotypes with basic chromosome numbers of *x* = 5, *x* = 6, and two with *x* = 7, have so far been described [24].

The two *x* = 7 diploid cytotypes are referred to as AA and B^7^B^7^, with AA found only in countries bordering the Atlantic Ocean in Iberia and North Africa and B^7^B^7^ occupying the countries around the Mediterranean Basin and on its islands; they overlap only in Spain [25]. The karyotype morphologies of AA and B^7^B^7^ are very similar, but differ significantly in chromosome size and DNA amount, and, more trivially, in the location of the single NOR within chromosome 3 [25,26,34]. Cytotype B^7^B^7^ has been hypothesized to be most similar to the ancestral karyotype of the complex [26].

Diploid plants based on *x* = 6 (cytotype B^6^B^6^) are endemic to Crete. The B^6^B^6^ karyotype carries a large submetacentric chromosome referred to as F^1^(6–7) [F = fusion/fission; numbers in parentheses indicate chromosomes proposed to be involved in fusion/fission], while the remaining chromosomes correspond closely in morphology and homoeology to chromosomes 1–5 of the B^7^/A genomes. A diploid of constitution B^5^B^5^ is endemic to Libya [26] and carries two fission/fusion chromosomes, assigned to as F^2^(6–7) and F^3^(1–3), with respect to the karyotype of A/B^7^ genome [24,26].

Despite the enormous chromosomal and DNA amount variation within the *P. autumnale* complex, there is no large-scale accompanying morphological differentiation. The mechanisms involved in chromosome change and its directionality, might therefore allow us to infer evolutionary patterns within the genus. Within *P. autumnale*, we have previously [24,35,36] attempted to establish phylogeny from chromosome numbers and karyotype structure supplemented by analyses of meiotic configurations in diploid hybrids. Two sequential chromosomal fusions were proposed for the reduction of the chromosome number from *x* = 7 (AA, B^7^B^7^) to *x* = 6 (B^6^B^6^) and *x* = 5 (B^5^B^5^) [26]. In addition to this descending dysploid shift, genome size also varies, with a major discontinuity between genomes B^7^, B^6^ and B^5^ and the large genome A [26,37]. No evolutionary directionality has been ascribed to this change.

Phylogenetic relationships within the family Hyacinthaceae have been inferred from plastid DNA sequence analyses [38-40]. These studies, however, included only one or two accessions of *Prospero* (of unknown ploidy levels), so no assessment of phylogenetic relationships within the genus could be made. This present study provides the first comprehensive analysis of phylogenetic relationships among all the diploids identified in the genus *Prospero*, based on karyotype and genome size changes, analyzed against a rigorous DNA phylogeny, allowing previous hypotheses concerning karyotypic evolution to be tested. This study provides also a framework for studying evolutionary patterns in polyploid genomes of *Prospero*.

The aims of this study are to: (1) establish numbers and locations of 5S and 35S rDNA loci in all diploid species and cytotypes of *Prospero;* (2) analyze the evolution of rDNA loci and genome size in a phylogenetic context; (3) test previous hypotheses concerning the evolution of basic chromosome number in the *P. autumnale* complex; and (4) propose a new model for chromosomal rearrangements within the genus and to evaluate their role in the diversification of taxa.

## Results

### Chromosome numbers and karyotype structure in the genus *Prospero*

**Figure 2:**
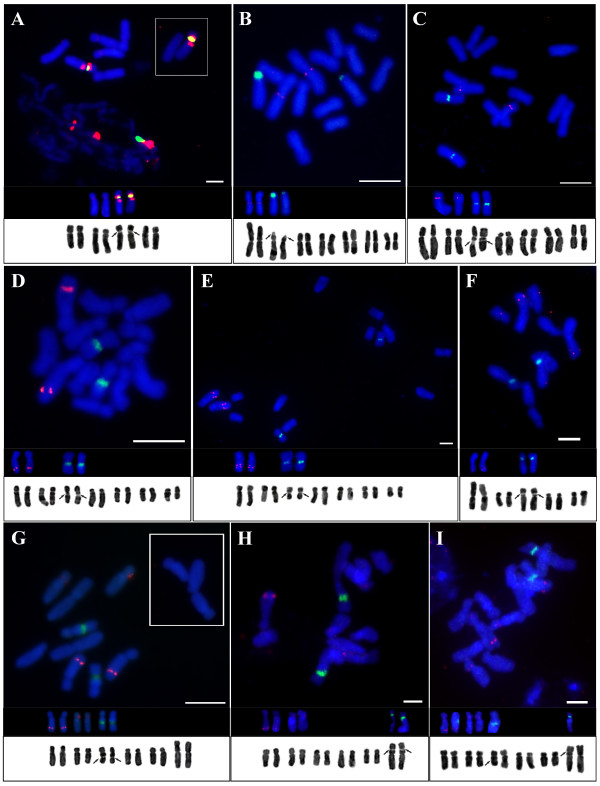
**Karyotypes and localization of 35S (green) and 5S (red) rDNA loci in diploids of *Prospero*. (A)***P*. *obtusifolium*; **(B)***P*. *hanburyi*; **(C–I)***P. autumnale* complex: **(C)** AA; **(D)** B^7^B^7^; **(E)** B^7^B^7^ with duplicated 5S rDNA locus in chromosome 1; **(F)** B^5^B^5^; **(G)** B^6^B^6^; **(H)** B^6^B^6^ with homozygous NOR translocation (NOR in pair of chromosome 6); **(I)** B^6^B^6^ with heterozygous NOR translocation (NOR in one of each chromosome 3 and 6). Insets in **(A)** and **(G)** show chromosomes of a single cell which were lying at some distance from the main chromosome group and either could not be photographed together using high magnification objectives or were too far apart to clearly demonstrate chromosome morphology while showing the whole field. Scale bar = 5 μm.

Chromosome counts confirmed all chromosome numbers reported earlier for diploids in the genus *Prospero*: 2*n* = 8, 10, 12, and 14 (Table 1, Figure 2).

**Table 1:** Plant material studied with localities, chromosome numbers, and GenBank accession numbers of ITS DNA sequences

**Cytotype**	**Voucher information (accession number)**	**2*n***	**ITS GenBank accession number**
Outgroups			
*Dipcadi* sp.	cult. HBV (H336)	-	KC899267
*Othocallis siberica* (Haw.) Speta	cult. HBV (H2159)	12	KC899268
			
Genus *Prospero* Salisb.			
*P*. *obtusifolium* (Poir.) Speta	Spain, Parker s.n., cult. HBV (H540)	8	KC899275
	Morocco, Parker 15500–1, cult. HBV (H547)	8	KC899273
	Spain, Parker DL20, cult. HBV (H556)	8	KC899276
	Spain, Parker DL8, cult. HBV (H559)	8	KC899272
	Morocco, Parker 15607, cult. HBV (H563)	8	KC899277
	Morocco, Parker 15607, cult. HBV (H564)	8	KC899274
*P*. *hanburyi* (Baker) Speta	Turkey, Findikpinar A, Leep s.n., cult. HBV (H115)	14	KC899269
	Turkey, Narlikuyu, Silifke, 475/01, cult. HBV (H231)	14	KC899270
	Turkey, Findikpinar, L75/T25, cult. HBV (H397)	14	KC899271
*P*. *autumnale* (L.) Speta s.l.			
AA	Spain, Huelva, Parker s.n., cult. HBV (H541)	14	KC899278
	Spain, Badajoz, Parker CV3, cult. HBV (H543)	14	KC899279
	Spain, Huelva, Parker s.n., cult. HBV (H548)	14	KC899280
	Portugal, Peniche, Parker s.n., cult. HBV (H550)	14	KC899281
	Portugal, Peniche, Parker s.n., cult. HBV (H551)	14	KC899283
	Spain, Huelva, Parker s.n., cult. HBV (H557)	14	KC899282
B^7^B^7^	Greece, Crete, Speta KR245, cult. HBV (H47)	14^1^	KC899309
	Greece, Peloponnisos, Speta 81, cult. HBV (H74)	14^1^	KC899308
	Greece, Rhodos, Faliraki, Speta 52800, cult. HBV (H137)	14^2^	KC899296
	Höner, s.n., cult. HBV (H228)	14^2^	KC899295
	Cyprus, Speta 53872, cult. HBV (H239)	14^1^	KC899297
	Greece, Samos, Tod 52684, cult. HBV (H241)	14^1^	KC899310
	Montenegro, Speta s.n., cult. HBV (H422)	14^2^	KC899302
	Montenegro, Speta s.n., cult. HBV (H424)	14^2^	KC899305
	Italy, Sicily, Speta 51990, cult. HBV (H428)	14^1^	KC899298
	Greece, Crete, Speta KR 15, cult. HBV (H440)	14^1^	KC899306
	Speta 52746, cult. HBV (H447)	14^1^	KC899299
	Greece, Kalamitsi, Speta 52690, cult. HBV (H450)	14^1^	KC899311
	Greece, Crete, Speta s.n., cult. HBV (H460)	14^1^	KC899307
	Greece, Naxos, Speta 3, cult. HBV (H575)	14^1^	KC899300
	Serbia, Siget-Baun, Rat s.n., cult. HBV (H576)	14^2^	KC899303
	Ukraine, Nikita, Roman RK4-1, cult. HBV (H591)	14^2^	KC899304
	Israel, Nene Han, Parker, s.n., cult. HBV (H612)	14^1^	KC899301
B^6^B^6^	Greece, Crete, Speta KR20, cult. HBV (H158)	12	KC899289
	Greece, Crete, Speta CR95-99, cult. HBV (H166)	12	KC899284
	Greece, Crete, Speta 95–99, cult. HBV (H170)	12	KC899285
	Greece, Crete, Speta KR20, cult. HBV (H195)	12	KC899290
	Greece, Crete, Jahn 854, cult. HBV (H197)	12^4^	KC899286
	Greece, Crete, Speta 52635, cult. HBV (H274)	12	KC899291
	Greece, Crete, N.B. 6890, cult. HBV (H340)	12^3^	KC899287
	Greece, Crete, Jahn 353, cult. HBV (H408)	12	KC899288
	Greece, Crete, Jahn & Böhling 9131Z, cult. HBV (H427)	12	KC899293
	Greece, Crete, Speta CR95-99, cult. HBV (H468)	12	KC899292
	Greece, Crete, Speta 52613, cult. HBV (H520)	12	KC899294
B^5^B^5^	Libya, Mt. Tobi, Parker s.n., cult. HBV (H566)	10	KC899313
	Libya, Mt. Tobi, Parker To-2, cult. HBV (H581)	10	KC899314
	Libya, Mt. Tobi, Parker To-28, cult. HBV (H582)	10	KC899316
	Libya, Mt. Tobi, Parker s.n., cult. HBV (H631)	10	KC899317
	Libya, Mt. Tobi, Parker s.n., cult. HBV (H637)	10	KC899312
	Libya, Nagasa, Parker s.n., cult. HBV (H640)	10	KC899315

^1^One locus of 5S rDNA.

^2^Duplication of 5S rDNA locus in chromosome 1.

^3^Translocation of NOR of one on the homologous chromosomes 3 to chromosome F^1^(6–7).

^4^Translocation of both NORs to chromosome F^1^(6–7).

Plant material is in cultivation in Botanical Garden of the University of Vienna (HBV). Each individual in cultivation has a unique ID (in brackets, e.g., H336).

#### Prospero obtusifolium

All six plants of *P. obtusifolium* were diploid with 2*n* = 2*x* = 8 (Table 1, Figure 2). The karyotype consisted of three pairs of submetacentric and one pair of sub-telocentric chromosomes (Figure 2) with Haploid Karyotype Length (HKL) of 29.01 ± 0.77 μm (Table 2). A single nucleolar-organizing region (NOR) was localized within the pericentric region of the short arm of chromosome 3 (Figure 2). The 1*C* DNA amount of *P. obtusifolium* was 4.94 ± 0.039 pg (Table 2).

**Table 2:** **Genome size, karyotype length and rDNA loci number and localization in *Prospero***

**Cytotype**	**5S and 35S rDNA loci number and localization**^**1, 2**^		**Genome size**	**Chromosome size**
**(Accession number)**			**1C (pg) ± SD**	**HKL (μm) ± SD**
*Prospero hanburyi*				
(H397)	5S (L-P^chr1^)		6.81 ± 0.017	44.90 ± 4.04
	35S (S-ST^chr2^)			
*P*. *obtusifolium*				
(H540)	5S (L-P^chr2^, L-P^chr3^, S-P^chr3^)		4.94 ± 0.039	29.01 ± 0.77
	35S (S-P^chr3^)			
*P*. *autumnale* complex				
AA				
(H551)	5S (S-P^chr2^)		7.85 ± 0.045	48.35 ± 7.15
	35S (L-P^chr3^)			
B^7^B^7^				
(H450)	5S (L-D^chr1^)		4.23 ± 0.048	33.76 ± 1.45
	35S (L-P^chr3^)			
(H424)	5S (L-D^chr1^)		4.45 ± 0.023	28.70 ± 1.74
	35S (L-P^chr3^)			
B^6^B^6^				
(H274)	5S (L-D^chr1^, S-P^chr2^)		6.27 ± 0.083	38.34 ± 1.24
	35S (L-P^chr3^)			
B^5^B^5^				
(H640)	5S (L-D^chr1^)		4.86 ± 0.002	29.67 ± 2.58
	35S (S-P^chr3^)			
Translocations				
B^6^B^6^	5S (L-D^chr1^, S-P^chr2^)		6.05 ± 0.011	34.97 ± 3.98
(H197)	35S (S-P^chr6^)			
B^6^B^6^	5S (L-D^chr1^, S-P^chr2^)		6.07 ± 0.031	30.03 ± 1.99
(H340)^3^	35S (L-P^chr3^, S-P^chr6^)			

^1^ L, long arm; S, short arm; D, distal (interstitial) location of 5S rDNA; P, pericentric location of 5S or 35S rDNA; ST, subterminal location of 35S rDNA; ^chr^, number of the chromosome.

^2^Scale bar = 1 μm; only the chromosomes bearing rDNA are shown: filled and open circles indicate position of 35S and 5S rDNA loci, respectively.

^3^Heterozygote; both chromosomes carrying 35S rDNA (chromosome 3 and chromosome 6) are shown.

#### Prospero hanburyi

The three plants of *P. hanburyi* were diploid with 2*n* = 2*x* = 14 (Table 1, Figure 2), comprising four pairs of near-metacentric and three pairs of submetacentric chromosomes (Figure 2). The HKL was 44.90 ± 4.04 μm (Table 2). A single NOR was localized subterminally on the short arm of chromosome 2 (Figure 2). This contrasted to the interstitial localization of NORs in all other *Prospero* taxa and cytotypes. The 1*C* content of *P. hanburyi* was 6.81 ± 0.017 pg (Table 2). The karyotypes of these two species showed little structural similarity to the diploid karyotypes within the *P*. *autumnale* complex (Figure 2).

#### The *Prospero autumnale* complex

The four diploid cytotypes (AA, B^5^B^5^, B^6^B^6^, and B^7^B^7^) of the *P*. *autumnale* complex differred not only in basic chromosome number, but also in karyotype structure due to fusion/fission and genome size (Table 1, Figure 2).

### Cytotype AA (2*n* = 2*x* = 14)

In all six individuals the karyotype consisted of five sub-metacentrics (chromosomes 1–3 and 5–6), one sub-telocentric (chromosome 4), and one near-metacentric (chromosome 7; Figure 2). The HKL was 48.35 ± 7.15 μm (Table 2) with a 1*C* DNA content of 7.85 ± 0.045 pg (Table 2). A single NOR was adjacent to the centromere in the long arm of chromosome 3 (Figure 2).

### Cytotype B^7^B^7^ (2*n* = 2*x* = 14)

The karyotype in seventeen individuals consisted of five sub-metacentrics (chromosomes 1–3 and 5–6), one sub-telocentric (chromosome 4), and one near-metacentric (chromosome 7), each identifiable by size and morphology (Table 1 and Figure 2).

The karyotypes of A and B^7^ genomes were extremely similar in morphology and the numbers indicated homoeologies. HKL and 1*C* DNA contents have been established in selected individuals, which differed in their 5S rDNA locus number (for details see below). The HKLs were 28.70 ± 1.74 μm and 33.76 ± 1.45 μm while genome sizes were 4.45 ± 0.023 pg and 4.23 ± 0.048 pg respectively (Table 2). A single NOR was adjacent to the centromere in the long arm of chromosome 3 (Figure 2). Cytotype B^7^B^7^ is the most widespread in *P. autumnale.*

### Cytotype B^6^B^6^ (2*n* = 2*x* = 12)

**Figure 3:**
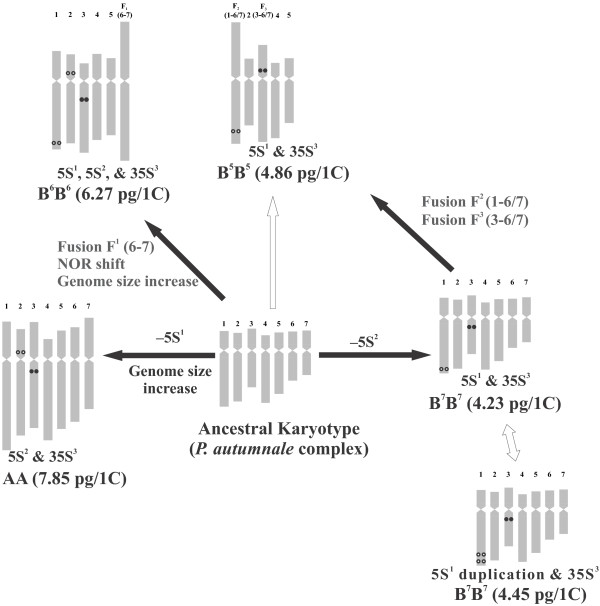
**Present hypothesis on genome evolution within *Prospero autumnale *complex.** The model is based on karyotype morphology, rDNA loci localization, and genome size interpreted in a phylogenetic context. 5S rDNA loci are indicated as open circles, and 35S rDNA loci as closed circles. Black arrows indicate more parsimonious hypotheses, empty arrows indicate alternatives.

In all eleven bulbs, the karyotype consisted of four sub-metacentrics (chromosomes 1–3 and 5), one sub-telocentric (chromosome 4), and one large sub-metacentric presumptive fusion chromosome classified as chromosome number F^1^(6-7). Chromosome numbering again reflects homoeology to B^7^ and A genomes (Figures 2 and 3, Additional file 1: Figure S1).

In nine individuals, both NORs were located in the long arm of the chromosome homoeologous to chromosome 3, although in a more median position (Figure 2). The other two individuals (from different populations) have apparently undergone translocation of one or both NOR regions, respectively. In one, the NORs were located in both homologues of chromosomes 6 (accession H197; Figure 2), and in the other in chromosome 3 and the same position on chromosome 6 (accession H340; Figure 2). The HKL of standard individuals was 38.34 ± 1.24 μm with a 1*C* DNA content of 6.27 ± 0.083 pg (H274; Table 2). The HKL of the NOR translocation heterozygote H340 was slightly lower and the genome size slightly smaller (30.03 ± 1.99 μm and 6.07 ± 0.031 pg; Table 2) while NOR translocation homozygote H197 had HKL of 34.97 ± 3.98 μm and genome size of 6.05 ± 0.011 pg (Table 2).

### Cytotype B^5^B^5^ (2*n* = 2*x* = 10)

In the six B^5^B^5^ individuals, the karyotype comprised two sub-metacentrics (chromosomes 2 and 5), one sub-telocentric (chromosome 4) (again reflecting homoeologies with B^7^ and A genomes), a large sub-metacentric fission/fusion chromosome F^2^(6–7), and a sub-metacentric fission/fusion chromosome F^3^(1–3) (Figure 2). In B^5^B^5^, the HKL was 29.67 ± 2.58 μm and the 1*C* DNA amount 4.86 ± 0.002 pg (Table 2). It has been proposed previously that B^5^B^5^ results from two fusions, one identical to that in the B^6^B^6^ karyotype (F^1^ = F^2^). The second fusion (F^3^) was more complex, but has been interpreted to be a result of chromosome 1 and 3 fusion (Additional file 1: Figure S1) relocating the NOR to the short arm of an enlarged fusion chromosome F^3^ (Additional file 1: Figure S1).

### 5S and 35S rDNA localisation

The three species of *Prospero* and all cytotypes invariably had one 35S rDNA locus per genome (Figure 2, Additional file 2: Figure S2). Its chromosomal localization was predominantly interstitial and adjacent to the centromere, except in *P. hanburyi* where it was sub-terminal. Either one or two 5S rDNA loci were found with a third, minor, locus in *P. obtusifolium* (Figure 2, Additional file 2: Figure S2). Locations of these loci were more variable than the 35S rDNA loci.

i. The 35S rDNA locus of *P. hanburyi* was sub-terminal on the short arm of chromosome 2, whereas the single 5S rDNA locus was located on the long arm of metacentric chromosome 1 adjacent to the centromere (Figure 2B).

ii. In *P. obtusifolium,* the 35S rDNA locus was on chromosome 3, flanked on each side by a 5S rDNA locus (Figure 2A). An additional minor 5S rDNA locus was seen occasionally, located on the long arm of chromosome 2 (Figure 2A).

iii. All cytotypes of *P. autumnale* possessed a single interstitial 35S rDNA locus, usually closely adjacent to a centromere. There were either one or two 5S rDNA loci in different cytotypes (Figure 2):

– in the AA cytotype, a single 5S rDNA locus was found in the pericentric region of the short arm of chromosome 2 (Figure 2C). The 35S rDNA locus was close to the centromere in the long arm of chromosome 3;

– cytotype B^7^B^7^ usually had a single 5S rDNA locus localized interstitially within distal region of the long arm of chromosome 1. Some individuals, however, had two loci in close proximity at this position, suggesting either local duplication of this chromosomal region or of the locus itself (Figure 2D–E). The single 35S rDNA locus was pericentromeric on chromosome 3 long arm (Figure 2D–E), in a similar location to that in AA (Figure 2C);

– cytotype B^6^B^6^ always had two 5S rDNA loci, a smaller one in the pericentric region of the short arm of chromosome 2 as in cytotype AA (Figure 2G) and a larger one in the distal region of the long arm of chromosome 1 as in B^7^B^7^. In most plants, there was a single 35S rDNA locus interstitial in the long arm of chromosome 3, although further from the centromere than that in AA and B^7^B^7^ (Figure 2C–E). Two plants differed from the standard pattern in their rDNA localization. In one individual, the 35S rDNA locus was close to the centromere in the short arm of submetacentric chromosome 6 (Figure 2H). In the other, one copy of the locus was detected in chromosome 6 and the other in the typical position on chromosomes 3 (Figure 2I).

– In the B^5^B^5^ cytotype, putative fusion chromosome F^2^(6–7) (Additional file 1: Figure S1) [26] had a 5S rDNA locus distal in one arm (Figure 2F). The 35S rDNA locus was localized interstitially close to the centromere within the short arm of the second-largest chromosome in the complement (Figure 2F), the putative fusion chromosome F^3^(1–3) (Additional file 1: Figure S1) [26].

### Phylogenetic relationships within *Prospero* based on ITS sequence data

**Figure 4:**
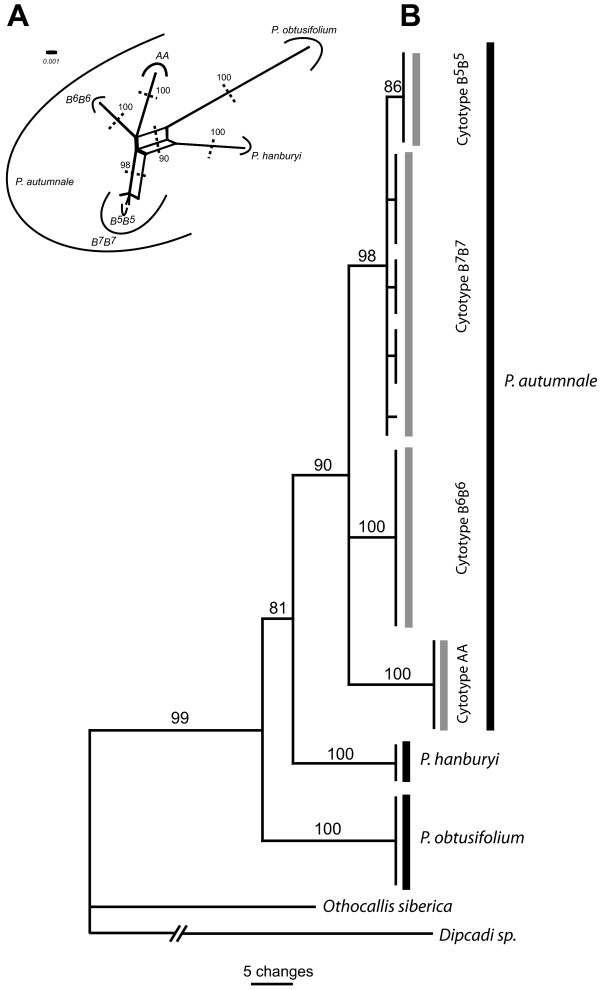
**Phylogenetic relationships within the genus *Prospero *inferred from ITS sequence data. (A)** NeighbourNet; **(B)** maximum parsimony phylogram.

Sequence analyses of ITS1 and ITS2 regions, including the intervening 5.8S coding region, of 35S rRNA have provided insights into the relationships amongst the diploids of *Prospero* (Figure 4). The length of the ITS region of the 49 analyzed diploid *Prospero* accessions ranged from 778 to 785 bp and the final, aligned dataset was 793 bp long. The maximum parsimony analysis of the ITS dataset resulted in four most parsimonious trees with a length of 216 steps (65 parsimony informative characters, consistency index [CI] = 0.926, retention index [RI] = 0.965, rescaled consistency index [RC] = 0.893). The final tree was rooted with two outgroup taxa (*Othocallis siberica* and *Dipcadi* sp., both in family Hyacinthaceae; Table 1). The genus *Prospero* was monophyletic (BS 99; Figure 4B). *P. obtusifolium* (6 individuals) and *P. hanburyi* (3 individuals) each formed well-supported clade (bootstrap support, BS 100). *P. obtusifolium* and *P. hanburyi* ITS regions differed by 29 substitutions, one of which is within one of two insertions (3 and 4 bp long) shared only by these two taxa (Additional file 3: Figure S3). *P. hanburyi* had an additional unique insertion of 3 bp. *P. hanburyi* and *P. obtusifolium* differed from B^7^B^7^ diploids by the above mentioned two shared insertions and by 13 and 28 substitutions, respectively (Additional file 3: Figure S3). *P. obtusifolium* (BS 100) was recovered as sister to clade comprising *P. hanburyi* and *P. autumnale* (BS 81). The *P. autumnale* complex formed a monophyletic and well-supported clade (BS 90). Within this clade cytotype AA (six individuals), formed a monophyletic sub-clade (BS 100; Figure 4). ITS sequences of all AA individuals were identical. ITS region of cytotype AA has two unique insertions (1 and 2 bp long, respectively; Additional file 3: Figure S3). The B^7^B^7^ cytotype (17 individuals) forms a well-supported clade (BS 98; Figure 4). This was the only cytotype within which ITS sequence variation has been observed (four distinct B^7^B^7^ groups, each having a unique substitution; Additional file 3: Figure S3). Interestingly, B^7^B^7^ clade includes all six individuals of the B^5^B^5^ cytotype nested within it (Figure 4A), or forming a sub-clade of unresolved relationship to B^7^B^7^ subclade with a bootstrap support of 86 (Figure 4B). All B^5^B^5^ individuals shared a unique 2 bp insertion compared with the B^7^ ITS sequence.

The B^6^B^6^ cytotype (2*n* = 2*x* = 12; eleven individuals) formed a well-supported monophyletic group (BS 100; Figure 4). The two B^6^B^6^ individuals with the 35S rDNA translocation did not show any ITS variation compared to other analyzed individuals. The ITS sequences of genomes B^6^ shared four unique substitutions (Additional file 3: Figure S3).

## Discussion

### Chromosome numbers and karyotype variation

The genus *Prospero* is highly variable in chromosome number and chromosome structure. Basic numbers have changed by dysploidy (*x* = 4, 5, 6, and 7) and, superimposed on this, high levels of auto- and allopolyploidy have evolved [24,26]. Three species are commonly recognized in the genus: *P*. *obtusifolium,* confined to the western Mediterranean islands and adjacent mainland, exclusively diploid with 2*n* = 8; *P*. *hanburyi* from the Levant, also a diploid but with 2*n* = 14; and the widespread *P*. *autumnale* complex with basic numbers of *x* = 5, 6, and 7 and an elaborate, reticulating auto- and allopolyploid series (from 3*x* to about 20*x,* but most frequently 4*x* and 6*x*; [24-26,28,29,34,37,41]).

Within the *P*. *autumnale* complex, four distinct cytotypes have been described and characterized so far [24,26]. A fifth genome, designated as B^7^* (or C), with chromosomes slightly smaller than B^7^ but of the same complement morphology, has so far been found only in allopolyploids on Crete [26]. The diploid cytotypes differ in chromosome number (2*n* = 2*x* = 10 [B^5^B^5^], 12 [B^6^B^6^], 14 [AA, B^7^B^7^]), in karyotype structure with one and two putative fusions resulting in B^6^ (F^1^) and B^5^ (F^2^ and F^3^) respectively, in NOR position, and in genome size, with a major difference in DNA amount between the A genome and the other three [26,37]. These studies are supported here, except that a few individuals with translocations were detected.

A combination of karyotypic features (chromosome size, morphology, NOR position, unique and stable locations of 5S and 35S rDNA loci, genome size) allows unambiguous identification of each cytotype as well as identification of homoeologous chromosomes between them (Figure 3). The karyotypes of *P. hanburyi* and *P. obtusifolium* differ from those of the *P. autumnale* complex to such an extent that it is impossible to infer any homoeologies between these taxa.

### Evolution of 5S and 35S rDNA loci

5S and 35S rDNA have been mapped to the chromosomes of all diploid species and cytotypes of the genus *Prospero.* This has allowed us to identify two and sometimes three chromosome pairs unambiguously (Figure 2). Thus, despite the high frequency of chromosomal rearrangements within and between populations of the *P. autumnale* complex - inversions, supernumerary segments, translocations, B-chromosomes [24,25,37,42] - all diploid cytotypes possess unique and stable locations of their rDNA loci (except for two B^6^B^6^ plants in this study with translocations). By contrast 5S rDNA proved to be more variable in locus number and location (Figure 2), a phenomenon observed in other plant groups [5,15,16,43,44]. Despite this variability, 5S rDNA is frequently more stable in its position than 35S rDNA, which may vary substantially in distribution between related species (e.g., in *Aloe*[45]) and even between cells in individuals of species of genus *Allium*[46].

*P. obtusifolium* exhibits a remarkable pattern of rDNA distribution, unique within *Prospero*: juxtaposition of a centromere and the 35S rDNA locus, with a 5S rDNA locus on each side (Figure 2A). Co-location of 35S and 5S rDNA within the same chromosome or chromosomal arm has, however, been reported in other plant groups [16,47-49], sometimes even as 5S rDNA units inserted within 35S rDNA repeats [17].

Within Hyacinthaceae, genera related to *Prospero* possess basic numbers of *x* = 7 or higher [28]. *Prospero obtusifolium* forms the basal clade in the ITS-derived phylogeny. It probably represents an old segregate within the genus, which is estimated to be 6.43 Ma old [40], and has experienced chromosomal rearrangements leading to a drastic chromosome number reduction to *x* = 4.

*P*. *hanburyi* is the only species in the genus to possess a subterminally localized 35S rDNA locus, instead of interstitial secondary constrictions adjacent to centromeres. It has been argued that a subterminal position for 35S rDNA is ancestral [50], but in *Prospero* it might also be associated with a high potential of 35S rDNA for generating chromosomal translocations [51,52]. The single 5S rDNA locus is located in unique chromosomal position close to the centromere of chromosome 1. It shares a common ancestry with the 5S rDNA locus of chromosome 1 in *P. autumnale*, as indicated by the phylogenetic analyses of the non-transcribed spacer region (K. Emadzade, H. Weiss-Schneeweiss et al., unpublished observations).

In contrast to the other two species, the diploids of the *P. autumnale* complex lend themselves to comparative karyotype analysis, due to the well-preserved chromosomal homoeology during evolution. Homoeology was first demonstrated in A and B^7^ diploid homoploid hybrids [35,36], and was extended to B^7^, B^6^ and B^5^ by analyses of meiotic pairing in diploid hybrids ([26,34], discussed below). The position of 35S rDNA is relatively conserved in the complex: within the long arm of chromosome 3, except when affected by the fusion in cytotype B^5^B^5^. The NOR chromosome (3) in the B^6^ genome has a similar size and arm ratio to chromosome 3 in B^7^, but it differs in the proximity of the NOR to the centromere, probably as a result of paracentric inversion (“NOR shift”, [24]). This regularity of interstitial position of 35S rDNA supports the earlier hypothesis [51] that it might provide greater karyomorphological stability during race or species evolution. The 5S rDNA loci are either interstitial in the distal part of the long arm of submetacentric chromosome 1 and/or proximal in the short arm of submetacentric chromosome 2, except where fusion has occurred in cytotype B^5^B^5^ (Figures 2 and 3). The only variation observed in the complex was a putative duplication of 5S rDNA locus in some copies of chromosome 1 of B^7^B^7^. Although phylogenetic analyses of ITS sequences did not ascribe any evolutionary significance to this duplication, phylogenetic analyses of the more variable 5S rDNA spacer (K. Emadzade, H. Weiss-Schneeweiss et al., unpublished observations) indicated that individuals carrying this duplication are more closely related to each other than to individuals carrying a single copy of the locus.

In addition to the between-cytotype variation in the number and distribution of 5S rDNA loci, variation in FISH signal intensity has frequently been observed (e.g., in the B^6^B^6^ cytotype; Figure 2G). Signal strength differences are likely to be correlated with copy-number variation at the target site [53].

### Phylogenetic interpretation of chromosomal variation in *Prospero*

The phylogeny of *Prospero,* inferred from ITS sequences, strongly supports monophyly of each species and diploid cytotype. *P. obtusifolium* and *P. hanburyi* are always recovered as subsequent sister groups to the *P. autumnale* complex. Neither species, however, has obvious chromosome homoeology with *P. autumnale*. By contrast, the ITS phylogeny coupled with knowledge of chromosome numbers, karyotype structure, and genome size allows us to test previous hypotheses concerning the direction and mechanisms of karyotype evolution within the *P. autumnale* complex (Figure 3, Additional file 1: Figure S1).

We offer a modified and more detailed model of the chromosomal changes involved in the origin of the cytotypes (Figure 3). Each cytotype forms a well-supported clade, with cytotype AA being the most distinctive. Cytotype AA is found only in the western distribution area of the genus, adjacent to the Atlantic Ocean and might have been isolated by a Pleistocene glacial advance.

The ITS phylogeny supports the origin of cytotype B^5^B^5^ from B^7^B^7^ rather than from B^6^B^6^, with genome B^6^ being sister to B^7^. The close relationship of the localized cytotype B^5^B^5^ within the widespread cytotype B^7^B^7^ (Figure 4A) suggests its recent origin, and that it is the youngest segregate of the complex. Intra-cytotype ITS sequence variation has only been observed within the widespread cytotype B^7^B^7^. This contrasts with a lack of variation in all other more geographically localized or endemic cytotypes and species. Thus phylogenetic and chromosomal data, and particularly the distribution of 5S rDNA loci in cytotypes B^5^B^5^, B^6^B^6^, and B^7^B^7^, suggest independent and not sequential dysploidy: from *x* = 7 to *x* = 6 and independently from *x* = 7 to *x* = 5.

### Model of karyotype evolution in *Prospero autumnale* complex

Genome size estimations presented in the current study (Table 2, Figure 3) differ from genome size measurements published previously (Additional file 1: Figure S1) [26]. These previous measurements have been performed using Feulgen densitometry which could account, at least partially, for the discrepancy. However, *Prospero* genome size measurements reported in another study [37] are largely congruent with our data.

The chromosome number and structure of the *Prospero* ancestral karyotype (genus-wide) remains obscure, as do the karyotype relationships of the three species (K. Emadzade, T.-S. Jang, H. Weiss-Schneeweiss et al., unpublished observations). The ancestral chromosome number of the *Prospero autumnale* complex has been inferred as *x* = 7, and this is also supported by phylogenetic reconstructions using extended plastid, ITS, and 5S rDNA spacer sequence datasets (K. Emadzade, H. Weiss-Schneeweiss et al., unpublished observations), with the ancestral karyotype similar in overall morphology to the A and B^7^ genomes [24,34] (Figure 3). These genomes each possess one 5S rDNA locus either in long arm of chromosome 1 (5S^1^; B^7^) or in the short arm of chromosome 2 (5S^2^; A). Sequencing of the NTS regions of these loci shows them to be distinct (data not shown). We propose that the ancestral genome had both of these loci. The ancestral genome could have resembled A or B^7^ in size, or indeed be different from both, but increase is thought to be predominant to, and more rapid than, genome decrease. So resemblance of B^7^ to the ancestral karyotype is likely to be the most parsimonious, and genome increase might have occurred in the western refugium during a glacial maximum ([26], J. Parker, unpublished observations).

Loss of 5S rDNA from chromosome 1 (5S^1^) of the ancestral karyotype has likely accompanied evolution of cytotype AA. Its evolution has also been accompanied by nearly 70% genome size increase (Figure 3). Loss of the 5S^2^ rDNA locus from the ancestral karyotype would give rise to cytotype B^7^B^7^ (Figure 3), now widespread across the whole Mediterranean basin. Interestingly, seven of the seventeen B^7^ diploids analysed carried a duplication of the 5S^1^ rDNA locus.

Genome B^6^ may have originated from the ancestral karyotype with *x* = 7 by fusion of chromosomes 6 and 7 (Figure 3). It is also necessary to postulate a pericentric inversion and loss of a centromere in its evolution [26]. Previously, it had been proposed that the B^6^ genome evolved by chromosome fusion directly from B^7^ (Additional file 1: Figure S1). Evidence for the direct evolution from an ancestral karyotype rather than directly from B^7^ comes from the retention of both the 5S rDNA loci by B^6^. The analysis of meiotic pairing in hybrids does not differentiate between the two hypotheses [24,26]. The genome of B^6^ is 44% larger than B^7^, and the difference affects all chromosomes of the complement nearly equally. This is observed as bivalent asymmetry during meiosis in hybrids.

It was also proposed that B^5^ arose directly from B^6^ by a second fusion event [24]. The evidence came from the gross similarity of the largest fusion chromosomes in genomes B^6^ and B^7^ (thus they proposed F^1^ = F^2^), and the presence of two trivalents at meiosis in B^5^B^7^ hybrids. However, the molecular evidence presented here is consistent with B^5^ arising from B^7^, but supports evolution of B^6^ directly from an ancestral species of *P. autumnale*. The fusion chromosomes in B^5^ [F^2^(1-6/7), F^3^(3-6/7)] and B^6^ [F^1^(6–7)], therefore, have independent origins. No molecular markers are yet available to unequivocally identify chromosome 6 and 7, so the relationships of the fusion chromosomes cannot be explored more closely. The fusion chromosome F^2^ in cytotype B^5^ involving chromosome 1 and chromosome 6 or 7 [earlier proposed to be = to F^1^(6–7)], gives rise to a near-metacentric, the largest in the complement. As expected, this carries a 5S rDNA locus, which has been confirmed as 5S^1^ by sequencing (K. Emadzade, H. Weiss-Schneeweiss et al., unpublished observations). In addition, the genome size of B^5^ is 12% higher than B^7^ but it cannot be established at what point this may have occurred. The cytotype B^5^B^5^ is probably the most recently evolved diploid in the complex and is endemic to Libya, where it is the only race [34].

## Conclusions

Phylogenetic analysis has confirmed fusion and basic number reduction as opposed to fission and basic number increase as the evolutionary mechanisms characterizing karyotype evolution in the *P. autumnale* complex. Dysploidy has occurred twice via independent fusions, once perhaps ancestral from *x* = 7 to *x* = 6, and later a second time from *x* = 7 to *x* = 5. This extensive chromosomal evolution contrasts very strongly with a lack of morphological diagnostic features within the genus, which are particularly weak within the *P. autumnale* complex [29,33,54]. New species described in last few decades usually refer to small populations that differ mostly in quantitative characters, whose evolutionary significance needs to be evaluated using a more thorough sampling. Diversification and evolution of this genus, then, has occurred primarily through genome restructuring, with little involvement of morphological change. Genetic processes may, of course, be implicated in the generation of chromosomal change. Thus, the genus *Prospero*, and in particular the *P. autumnale* complex, provides a model system for studying the role of chromosomes in plant diversification.

This study of diploids in *Prospero* has laid foundations (1) to address the evolution of auto- and allopolyploidy within the complex which appear to follow different evolutionary trajectories; ([26,42], H. Weiss-Schneeweiss et al., unpublished observations), (2) to interpret the mechanisms involved in the origin and persistence of the many other types of chromosomal rearrangements that are found abundantly across the complex (such as B-chromosomes of many types, supernumerary segments on several chromosomes, translocations, and para- and pericentric inversions), and (3) to investigate the patterns of evolution of repetitive DNAs within the genus.

## Methods

### Plant material

Bulbs of all three *Prospero* species were collected from natural populations across the range (Table 1, Figure 1) and grown in the Botanical Garden of the University of Vienna. Due to high level of chromosomal variation, all individual bulbs were karyotyped prior to the FISH and phylogenetic analyses to select diploids (603 bulbs in total; T.-S. Jang, H. Weiss-Schneeweiss, unpublished observations). Where possible at least five bulbs with a “standard” (most common, without structural polymorphisms) karyotype were selected for the analyses; only three individuals with healthy root tips were available in *P. hanburyi*. *Othocallis siberica* and *Dipcadi* sp. (both in family Hyacinthaceae) were used as outgroup in phylogenetic analyses.

### Karyotype analysis and fluorescence *in situ* hybridization (FISH)

Actively growing root-tip meristems were pretreated with 0.05% aqueous solution of colchicine for 4 h at room temperature, fixed in ethanol : acetic acid (3 : 1) for at least 3 h at room temperature, and stored at −20°C until use.

Chromosome counting and basic karyotype analyses were performed using the standard Feulgen staining technique [55]. Ideograms (Additional file 2: Figure S2) were constructed based on measurements of at least five well-spread metaphase plates per individual (not shown) and measurements were used to calculate Haploid Karyotype Length (HKL). A single ideogram of each species and cytotype is provided, except for cytotypes B^7^B^7^ and B^6^B^6^ in which structural chromosomal variants were found (Table 2). Idiograms were constructed using Autoidiogram software (courtesy of Dr Wolfgang Harand, formerly University of Vienna; for details see [55]).

Chromosomal spreads for FISH were prepared by enzymatic digestion and squashing, as described earlier [4,16] with some modifications. Briefly, material was digested with 1% cellulase Onozuka (Serva, Heidelberg, Germany), 1% cytohelicase (Sigma-Aldrich, Vienna, Austria), and 1% pectolyase (Sigma-Aldrich) for 18 min at 37°C. Cover slips were removed at −80°C and preparations air-dried. FISH followed the established protocol [16,56]. Probes used for FISH were: 35S (18S/25S) rDNA from *Arabidopsis thaliana* in plasmid pSK+; 5S rRNA genic region from *Melampodium montanum* in plasmid pGEM-T Easy. Probes were labeled with biotin or digoxygenin (Roche, Vienna, Austria) either directly by PCR (5S rDNA) or using a nick translation kit (35S rDNA; Roche, Vienna, Austria). Digoxygenin was detected with antidigoxygenin antibody conjugated with FITC (5 μg mL^-1^: Roche, Vienna, Austria) and biotin with ExtrAvidin conjugated with Cy3 (2 μg mL^-1^: Sigma-Aldrich, Vienna, Austria). Preparations were analyzed with an AxioImager M2 epifluorescent microscope (Carl Zeiss, Vienna, Austria), images captured with a CCD camera, and processed using AxioVision ver. 4.8 (Carl Zeiss, Vienna, Austria) with only those functions that apply equally to the whole image. For rDNA localization, a minimum of 20 well-spread metaphases and prometaphases was analysed for each individual.

### DNA amplification, sequencing, and phylogenetic approach

Total genomic DNA was extracted from silica-dried leaf material using the standard CTAB procedure [57] with some modifications [58]. The nuclear ITS region (partial 18S rRNA gene, ITS1, 5.8S rRNA gene, ITS2, and partial 25S rRNA gene) was amplified with universal primers (ITS 18 s F and ITS 26 s R, [59]).

Polymerase chain reactions were carried out using 0.4 mM of each primer, ReddyMix (Abgene, Vienna, Austria) including 2.5 mM MgCl_2_ and 4% (v/v) dimethyl sulfoxide (DMSO). All PCR reactions were performed on an ABI thermal cycler 9700 (Applied Biosystems, Foster City, CA, USA) with the initial 3 min at 95°C, followed by 30 cycles each of 30 s at 96°C, 30 s at 58°C, and 2 min at 72°C, followed by a final elongation at 72°C for 8 min. Amplified fragments were checked on 1% (w/v) agarose gel and purified using exonuclease I (ExoI) and calf intestine alkaline phosphatase (CIAP) according to the manufacturer’s protocol (Fermentas, St. Leon-Rot, Germany). The purified fragments were directly sequenced using the PCR primers and dye terminator chemistry following the manufacturer’s protocol (Applied Biosystems). Sequencing reactions were run on a 48-capillary sequencer (3730 DNA Analyzer, Life Technologies). Sequences were assembled in SeqManII (Lasergene, Madison, WI) and manually aligned in BioEdit software ver. 7.0.5.3 [60]. Indels were coded as binary characters following the “modified complex coding method” [61] using the program SeqState version 1.36 [62], and the dataset with coded gaps was used for all analyses. A heuristic search for most parsimonious (MP) trees was performed using PAUP 4.0.b10 [63]. The analyses involved 1000 replicates of random sequence addition, with tree bisection–reconnection (TBR) and branch swapping, saving no more than 10 trees per replicate. All characters were equally weighted and treated as unordered. Strict consensus trees were computed from all equally most parsimonious trees. Internal branch support was estimated using non-parametric bootstrapping [64] with 10 000 replicates and 10 addition sequences replicates. Neighbor Net implemented in SplitsTree4 v. 4.11.3 [65], with gaps and ambiguous sites treated as missing data, was used to create the ITS network. Split support was calculated with 1000 bootstrap replicates. All ITS sequences are deposited in GenBank (accession numbers provided in Table 1) and the alignment and phylogeny are deposited in treeBASE (submission number 14243).

### Genome size estimation by flow cytometry (FCM)

The 1*C* values of all *Prospero* species and each cytotype of *P. autumnale* complex were measured using FCM with *Solanum pseudocapsicum* (1*C* = 1.29 pg, [66]) as the internal standard. Approximately 25 mg fresh leaves from each plant sample were co-chopped together [67] with standard material in Otto’s buffer I [68], and filtered through a 30 μm Nylon mesh. After 30 min RNase treatment at 37°C, the nuclei were stained in Otto’s buffer II [68] containing propidium iodide as the DNA stain. A CyFlow ML flow cytometer equipped with green laser (100 mW, 532 nm; Cobolt Samba; Cobolt AB, Stockholm, Sweden) was used for genome size estimation. The 1*C* values were calculated according to previously published formula [66].

## Availability of supporting data

Nucleotide sequences are available in GenBank (http://www.ncbi.nlm.nih.gov/genbank) under numbers KC899267-KC899317. Nucleotide alignment and phylogenetic analyses are deposited in treeBASE under study 14243 (http://purl.org/phylo/treebase/phylows/study/TB2:S14243).

## Competing interests

The authors declare that they have no competing interests.

## Authors’ contributions

TS-J carried out the cytogenetic studies, participated in the sequence alignment and drafted the manuscript. KE carried out sequencing of the ITS regions, sequence alignments, and phylogenetic analyses, and helped to draft the manuscript. JP provided plant material, participated in the design of the study and data interpretation, and helped to draft the manuscript. EMT carried out genome size measurements. ARL participated in the design of the study and data interpretation, and helped to draft the manuscript. FS provided plant material and helped to draft the manuscript. HW-S conceived of the study, and participated in its design and coordination and helped to draft the manuscript. All authors read and approved the final manuscript.

## Supplementary Material

Additional file 1: Figure S1 Previous hypothesis on karyotype evolution in the *Prospero autumnale* complex [26]. Black arrows indicate more parsimonious hypotheses, empty arrows indicate alternatives. Click here for file

Additional file 2: Figure S2 Ideograms of each of the standard (most frequent and without polymorphisms) diploid species and cytotypes analysed. Click here for file

Additional file 3: Figure S3 Alignment of variable nucleotide positions in the analysed ITS region. Click here for file
