# Supplementary figures and images for: Chromosomal diversification and karyotype evolution of diploids in the cytologically diverse genus Prospero (Hyacinthaceae)
Source: BMC Evol Biol. 2013 Jul 3;13:136. doi: 10.1186/1471-2148-13-136 (PMC3728210; doi:10.1186/1471-2148-13-136)

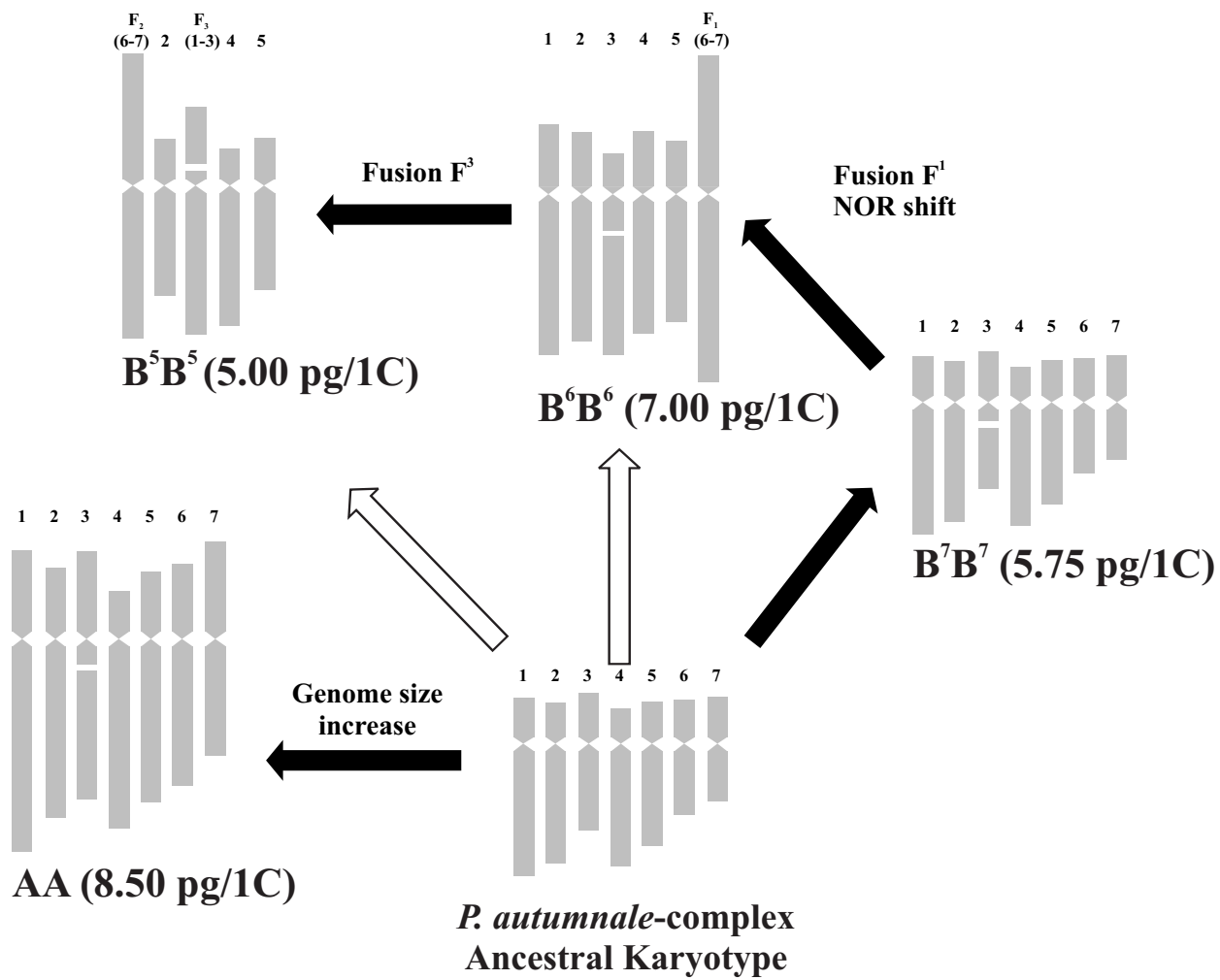

Supplement: Additional file 1: Figure S1 — Previous hypothesis on karyotype evolution in the Prospero autumnale complex [26]. Black arrows indicate more parsimonious hypotheses, empty arrows indicate alternatives. [file 1471-2148-13-136-S1.pdf]
