# Supplementary material for: Chromosomal diversification and karyotype evolution of diploids in the cytologically diverse genus Prospero (Hyacinthaceae)
Source: BMC Evol Biol. 2013 Jul 3;13:136. doi: 10.1186/1471-2148-13-136 (PMC3728210; doi:10.1186/1471-2148-13-136)

Figure S2. Ideograms of all analysed standard diploid species and cytotypes of the genus *Prospero.*
Green: localization of 35S rDNA loci; red: localization of 5S rDNA loci.

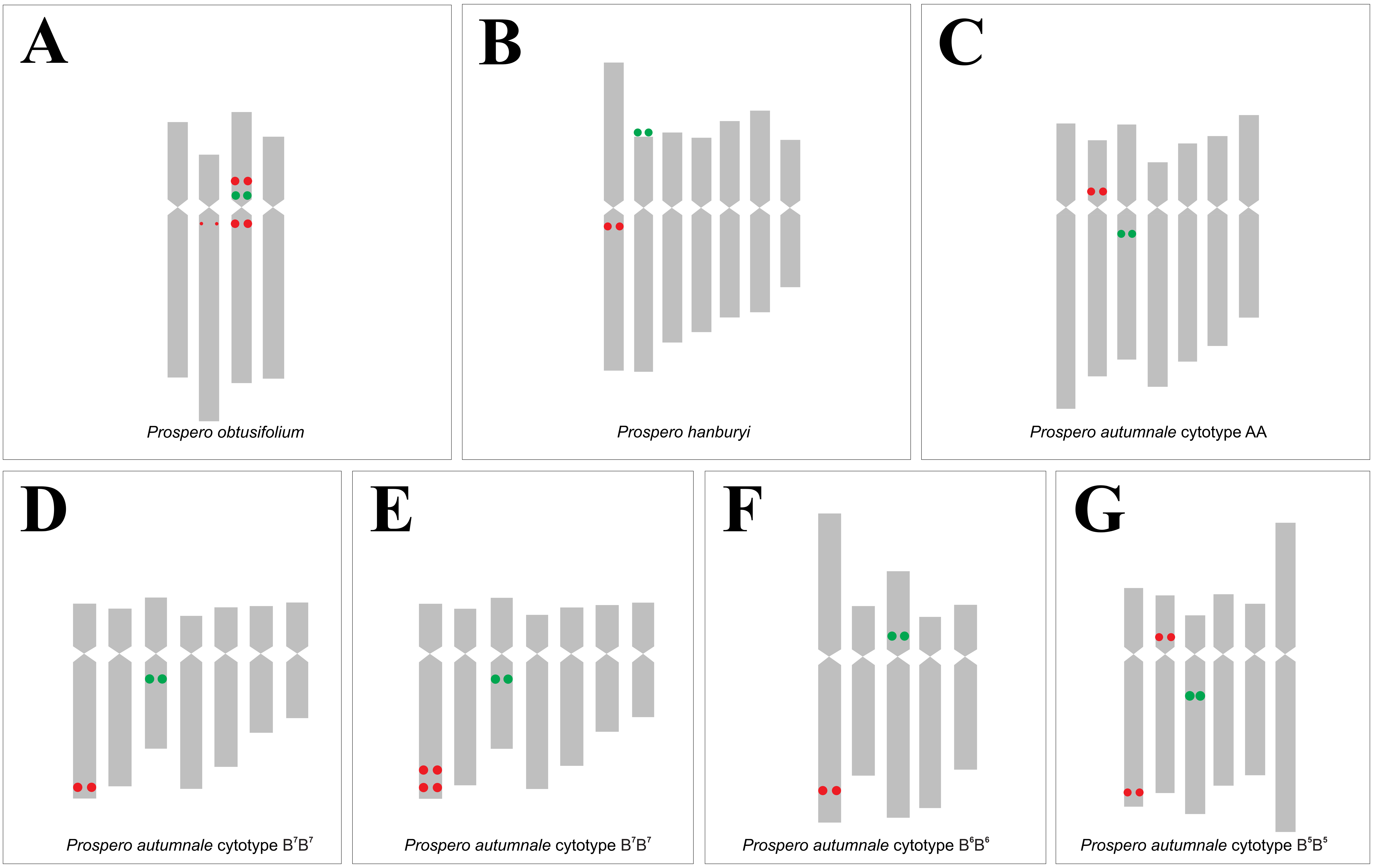

Supplement: Additional file 2: Figure S2 — Ideograms of each of the standard (most frequent and without polymorphisms) diploid species and cytotypes analysed. [file 1471-2148-13-136-S2.pdf]
