# Supplementary material for: Chromosomal diversification and karyotype evolution of diploids in the cytologically diverse genus Prospero (Hyacinthaceae)
Source: BMC Evol Biol. 2013 Jul 3;13:136. doi: 10.1186/1471-2148-13-136 (PMC3728210; doi:10.1186/1471-2148-13-136)
Supplement: Additional file 3: Figure S3 — Alignment of variable nucleotide positions in the analysed ITS region. [file 1471-2148-13-136-S3.pdf]

Supplementary Figure S3. Variable (parsimony informative) nucleotide positions in ITS DNA sequence alignment.

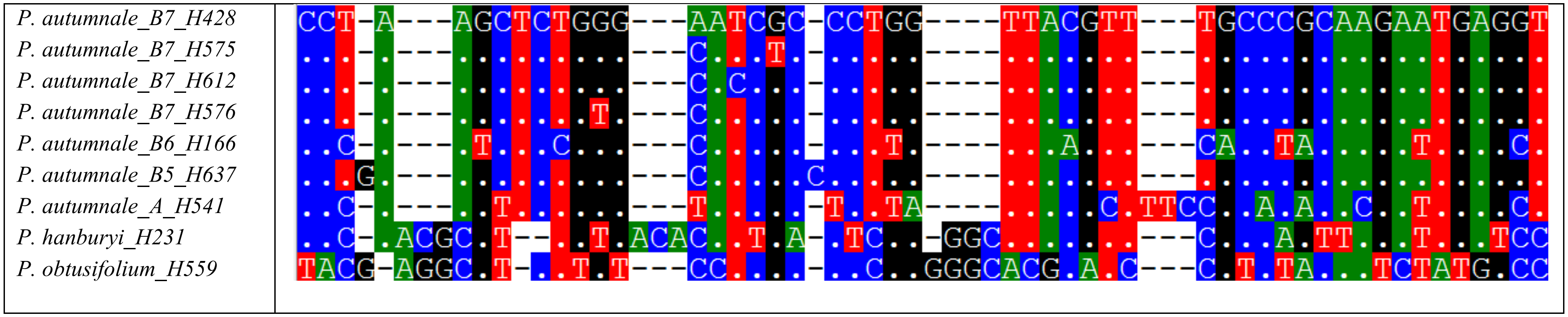

The following positions in alignment are shown: 2, 100, 107, 143, 144, 161, 162, 163, 174, 176, 183, 193, 194, 218, 225, 234, 235, 275, 276, 277, 284, 293, 297, 299, 300, 305, 306, 309, 311, 312, 320, 322, 324, 325, 326, 327, 339, 347, 348, 349, 351, 447, 524, 540, 547, 548, 559, 594, 598, 605, 616, 625, 626, 633, 641, 644, 647, 649, 661, 669, 680, 681, 720, 740.
